# Emergence activity at hibernacula differs among four bat species affected by white‐nose syndrome

**DOI:** 10.1002/ece3.9113

**Published:** 2022-07-13

**Authors:** Reilly T. Jackson, Emma V. Willcox, John M. Zobel, Riley F. Bernard

**Affiliations:** ^1^ Department of Biological Sciences University of Arkansas Fayetteville Arkansas USA; ^2^ Department of Forestry, Wildlife and Fisheries University of Tennessee Knoxville Tennessee USA; ^3^ Department of Forest Resources University of Minnesota St. Paul Minnesota USA; ^4^ Department of Zoology and Physiology University of Wyoming Laramie Wyoming USA

**Keywords:** bats, disease, hibernation behavior, PIT tags, winter activity

## Abstract

Prior to the introduction of white‐nose syndrome (WNS) to North America, temperate bats were thought to remain within hibernacula throughout most of the winter. However, recent research has shown that bats in the southeastern United States emerge regularly from hibernation and are active on the landscape, regardless of their WNS status. The relationship between winter activity and susceptibility to WNS has yet to be explored but warrants attention, as it may enable managers to implement targeted management for WNS‐affected species. We investigated this relationship by implanting 1346 passive integrated transponder (PIT) tags in four species that vary in their susceptibility to WNS. Based on PIT‐tag detections, three species entered hibernation from late October to early November. Bats were active at hibernacula entrances on days when midpoint temperatures ranged from −1.94 to 22.78°C (mean midpoint temperature = 8.70 ± 0.33°C). Eastern small‐footed bats (*Myotis leibii*), a species with low susceptibility to WNS, were active throughout winter, with a significant decrease in activity in mid‐hibernation (December 16 to February 15). Tricolored bats (*Perimyotis subflavus*), a species that is highly susceptible to WNS, exhibited an increase in activity beginning in mid‐hibernation and extending through late hibernation (February 16 to March 31). Indiana bats (*M. sodalis*), a species determined to have a medium–high susceptibility to WNS, remained on the landscape into early hibernation (November 1 to December 15), after which we did not record any again until the latter portion of mid‐hibernation. Finally, gray bats (*M. grisescens*), another species with low susceptibility to WNS, maintained low but regular levels of activity throughout winter. Given these results, we determined that emergence activity from hibernacula during winter is highly variable among bat species and our data will assist wildlife managers to make informed decisions regarding the timing of implementation of species‐specific conservation actions.

## Graphical abstract

Hibernating bats in eastern North America vary in their susceptibility to white‐nose syndrome, but mechanisms causing this variation are unclear. We investigated the relationship between activity during hibernation and susceptibility to white‐nose syndrome among four species of bats that vary in their susceptibility. We found that species with low susceptibility maintained regular activity levels throughout winter, albeit to different degrees. In comparison, medium‐high susceptibility species had pronounced activity levels during early and late hibernation.

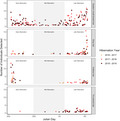

## 1 INTRODUCTION

Since its discovery in New York, United States (U.S.), in 2006, the disease white‐nose syndrome (WNS), caused by the fungal pathogen *Pseudogymnoascus destructans* (*Pd*), has spread through much of temperate North America causing extreme declines in several cave‐hibernating bat species (Cheng et al., 2021; Frick et al., 2017; Meteyer et al., 2009). While the disease is known to affect more than 10 North American species of bats, susceptibility to *Pd* invasion, WNS infection, and disease mortality seem to vary among them (Hoyt et al., 2021). Highly susceptible species, such as the little brown bat (*Myotis lucifugus*), northern long‐eared bat (*M. septentrionalis*), and tricolored bat (*Perimyotis subflavus*), are characterized by high *Pd* prevalence and fungal loads (i.e., infection intensity) within hibernating populations and have declined by 90% since the discovery of WNS (Bernard et al., 2017; Cheng et al., 2021; Frick et al., 2017; Langwig et al., 2015, 2016). In the U.S., northern long‐eared bats have been federally listed as threatened (U.S. Fish and Wildlife Service, 2016) and are pending elevation to endangered (U.S. Fish and Wildlife Service, 2022), with tricolored and little brown bats also petitioned for federal listing due to the disease (Center for Biological Diversity, 2010; Center for Biological Diversity and Defenders of Wildlife, 2016). The federally endangered Indiana bat (*M. sodalis*) has also experienced precipitous declines (84%), albeit not as severe as the aforementioned species (Cheng et al., 2021). In contrast, other cave‐hibernating species such as the eastern small‐footed bat (*M. leibii*) and the federally endangered gray bat (*M. grisescens*) exhibit low *Pd* prevalence and load and have not experienced dramatic population declines due to the disease (Bernard et al., 2017; Cheng et al., 2021; Frick et al., 2017; Hoyt et al., 2021; Langwig et al., 2015, 2016).

Multiple mechanisms influencing susceptibility to WNS are being explored, mainly in relation to hibernation ecology, torpor physiology, skin microbiota, and immune function (Hoyt et al., 2021). Periodic winter activity, one aspect of hibernation ecology, has been hypothesized to minimize susceptibility to *Pd* invasion and WNS infection (Bernard et al., 2017; Jackson et al., 2022; Moosman et al., 2015; Reynolds et al., 2017), as repeated arousals throughout hibernation may allow bats to groom more frequently, likely removing fungal conidia in the process and reducing *Pd* loads (Brownlee‐Bouboulis & Reeder, 2015). This episodic activity also raises body temperature, activating the immune system, and possibly slowing *Pd* growth, potentially resulting in milder infections (Prendergast et al., 2002; Dobony et al., 2011; Rowley & Alford, 2013; Verant et al., 2014). Furthermore, activities during arousals likely include drinking and foraging, which could help individuals combat some of the major effects of WNS infection, such as dehydration and starvation (Bernard et al., 2021; Cheng et al., 2019; Reynolds et al., 2017; Verant et al., 2014).

In the northeastern U.S. and Canada, winter emergence of bats from cave hibernacula and observations of dead bats on the landscape have been attributed to the effects of WNS (Blehert et al., 2009; Foley et al., 2011; Frick et al., 2016; Turner et al., 2011). However, in the southeastern U.S., a study completed in 2015 found that less than 50% of cave‐hibernating bat species captured outside of cave hibernacula during winter were positive for *Pd*, despite emerging from sites where *Pd* has been present for several years (Bernard et al., 2017). Additional evidence suggests that bats active on the landscape during winter are successfully foraging, acquiring energy intake that could aid in over‐winter survival (Bernard et al., 2017, 2021). Milder winters and the opportunity to forage could minimize fat depletion of bats infected with WNS, thus allowing populations of some species to rebound from the effects of the disease, especially when compared to hibernacula in more northern latitudes, where winters are longer and more severe and provide fewer opportunities for supplemental foraging (Bernard et al., 2017; Cheng et al., 2021; Grider et al., 2016).

The goal of our study was to explore winter activity, defined as flight activity at the entrance to or emergence from caves, of four target bat species (i.e., tricolored bat, Indiana bat, gray bat, and eastern small‐footed bat) with documented differences in susceptibility to WNS (high, medium, and low). We hypothesized that individuals within the medium‐to‐high susceptibility classification would become increasingly active throughout hibernation due to aberrant behavior caused by the increased fungal load. In contrast, we hypothesized that species with low susceptibility would be consistently active on the landscape during winter, where they can forage and activate immune function.

## 2 MATERIALS AND METHODS

### 2.1 Study area

**FIGURE 1:**
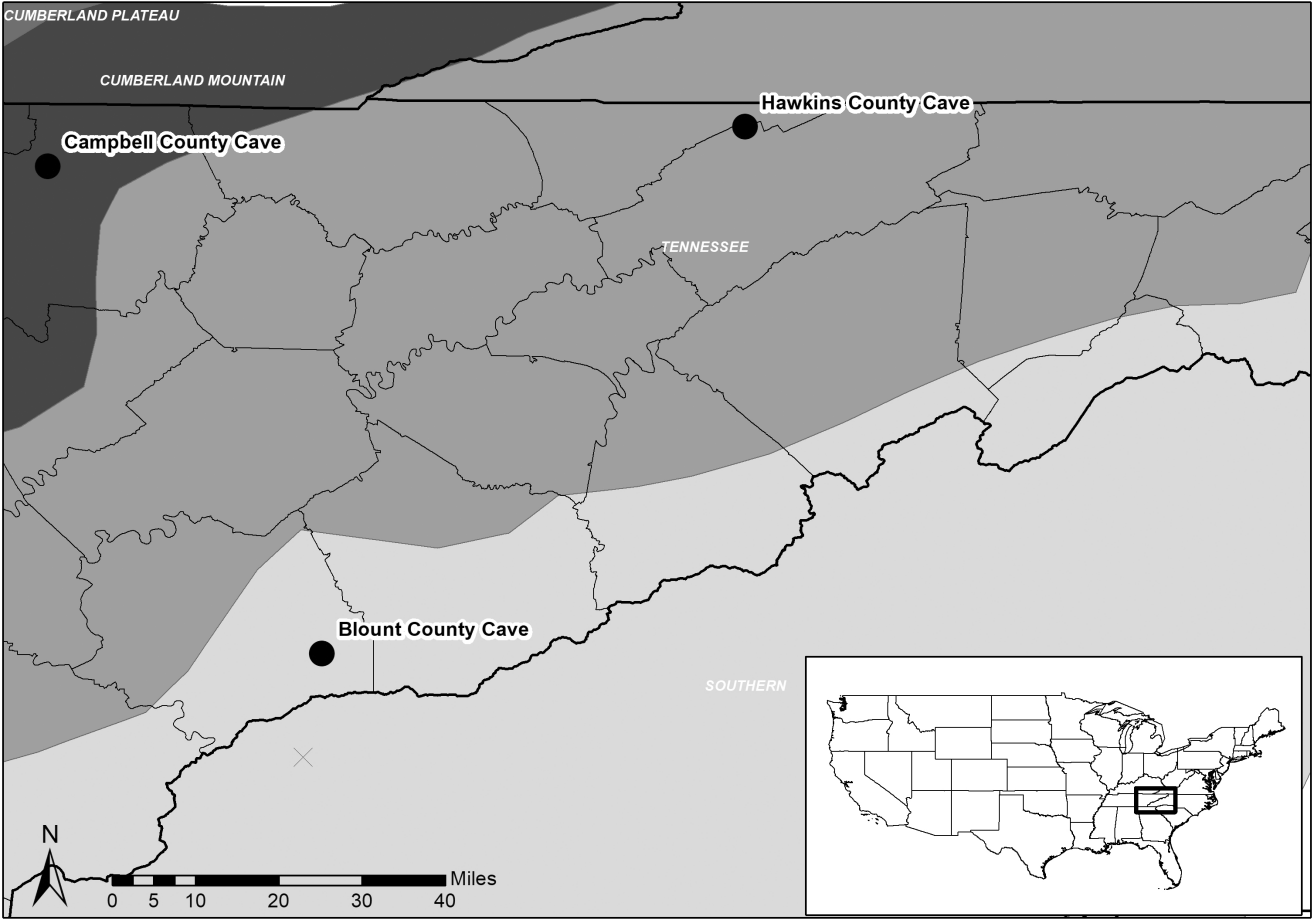
Three cave hibernacula in eastern Tennessee, U.S., where bats were captured and implanted with PIT (passive integrated transponder) tags. Passive integrated transponder tags were used to track bat movements as they entered and exited hibernacula during hibernation (November 1 to March 31), 2016–2019. Cave names were anonymized to ensure the protection of sensitive winter habitats and are shown within their respective physiographic regions

Our study was conducted at three cave hibernacula in eastern Tennessee, U.S., from 2016 to 2019 (Figure 1). All cave names have been anonymized with the county name to ensure the protection of sensitive habitats. Within our study period, mean daily temperatures during fall (October–November) ranged from 7 to 26°C with 0–12 days of minimum daily temperatures below 0°C (National Weather Service). The average daily temperature in winter (December–February) varied between 1.05 and 10.44°C with 6–26 days of minimum daily temperatures at or below 0°C. Finally, spring temperatures in March and April averaged between 9.17 and 23.17°C with 0–9 days recorded with minimum temperatures below 0°C. The temperatures during our study period were considered typical for this region (National Weather Service). Blount County Cave is in the Blue Ridge physiographic region and within the Great Smoky Mountains National Park, which is managed by the National Park Service (NPS). Prior to WNS, Blount County Cave was the largest known Indiana bat hibernaculum in the state. A population count conducted in February 2019 indicated Indiana bat numbers at the site had declined from »8000 to »750 individuals since WNS was confirmed in the winter of 2009/2010 (Campbell, 2019). Eastern small‐footed bats and tricolored bats are also encountered at Blount County Cave. Hawkins County Cave, managed by the Tennessee Wildlife Resources Agency (TWRA), is located within the Ridge and Valley physiographic region and is one of the largest gray bat hibernacula in the state, with an estimated population of over 350,000 individuals as of January 2019. White‐nose syndrome was confirmed at this site in the winter of 2011/2012 (Campbell, 2019). This cave also contains a small hibernating population of Indiana bats and low numbers of several other bat species. Finally, Campbell County Cave, managed by TWRA and The Nature Conservancy (TNC), is within the Cumberland Mountain physiographic region and contains approximately 1000 individuals, including »60 Indiana bats and 50–100 tricolored and eastern small‐footed bats. The presence of WNS was confirmed at Campbell County Cave during the winter of 2012/2013 (Campbell, 2019).

### 2.2 Assignment of WNS susceptibility

To determine the susceptibility designations of our focal species, we first defined the differences among high, medium, and low WNS susceptibility based on published findings from this region, WNS‐induced mortality among species, and our own findings from these study sites (Langwig et al., 2016; Bernard et al., 2017; Tables S1; and S2). High susceptibility was defined as average fungal loads of ≥ −2.5 log_10_ nanograms (ng) during winter, coupled with average prevalence (i.e., percent of infected bats captured) ≥80% (Bernard et al., 2017; Frick et al., 2017; Langwig et al., 2016). Medium susceptibility was defined as average fungal loads −4 to −2.5 log_10_ ng coupled with an average prevalence between 40 and 80%. Finally, we defined low susceptibility as average fungal loads < −4 log_10_ ng and average prevalence <40%. The species we examined with high susceptibility to WNS was the tricolored bat. We considered the Indiana bat to have medium‐to‐high susceptibility, meaning that this species exhibited medium fungal loads and high prevalence. Lastly, the eastern small‐footed and gray bat species were considered to have low susceptibility to WNS (Bernard et al., 2017; Frick et al., 2017; Langwig et al., 2012, 2016; Tables S1 and S2).

### 2.3 Bat capture and tagging

During fall swarming (September 1 to October 31) and spring emergence (April 1 to April 30) of 2016–2019, we used mist nets (Avinet Inc., Dryden, NY; mesh diameter: 75/2, 2.6 m high, 4 shelves, 4–9 m wide) and harp traps (Bat Conservation and Management, Inc., Carlisle, PA) to capture bats flying at cave entrances 1–3 times per week. We captured bats on nights when there was no precipitation and temperatures were above 0°C. We opened mist nets and harp traps 30 minutes before civil sunset and left them open for 2–5 h, or until temperatures fell below 0°C. Bats were captured at Blount County Cave from fall swarming of 2016 until fall swarming of 2019. Capture attempts at Hawkins County Cave were initiated during the fall swarming period of 2016 until the spring staging period of 2018. Finally, at Campbell County Cave, bats were captured beginning in the spring emergence period of 2017 until the fall swarming period of 2019.

Upon capture, bats were held for no longer than 30 min in individual bags in an insulated cooler with a heat source (HotHands®, Dalton, Georgia, U.S.). For all individuals captured, we recorded age, sex, right forearm length (mm), and body mass (g). Each bat captured was fitted with a unique forearm band (Porzana, Ltd., Icklesham, East Sussex, UK), with eastern small‐footed and tricolored bats receiving 2.4 mm bands and gray and Indiana bats receiving 2.9 mm bands. To determine bat emergence activity during hibernation (November 1 to March 31), we implanted bats with a uniquely identifiable 12 mm passive integrated transponder (PIT) tag (HDX12 Preloaded, Biomark, Inc., Boise, ID). We implanted PIT‐tags subcutaneously, just below the shoulder blades in the interscapular region (Britzke et al., 2014; Johnson et al., 2012; O'Shea et al., 2010) using single‐use needles and an implant gun (MK25, Biomark, Inc., Boise, ID). We only implanted PIT tags in individuals with a body mass ≥4.0 g and with no obvious health issues or injuries to minimize the stress on bats preparing for hibernation. All bats were then released at the site of capture.

While mist netting and harp trapping, we followed decontamination procedures outlined by the U.S. Fish and Wildlife Service (Shelley et al., 2013). Capture, handling, sample collection, and PIT‐tagging protocols were approved by the University of Tennessee Institutional Animal Care and Use Committee (IACUC 2253‐0317) and authorized under scientific collection permits from the USFWS (TE35313B‐3), NPS (GRSM‐2018‐SCI‐1253), TWRA (3742), and Tennessee Department of Environment and Conservation (2009‐038). Handling techniques were conducted as described by the American Society of Mammalogists (Sikes et al., 2016).

### 2.4 Emergence activity frequency

**FIGURE 2:**
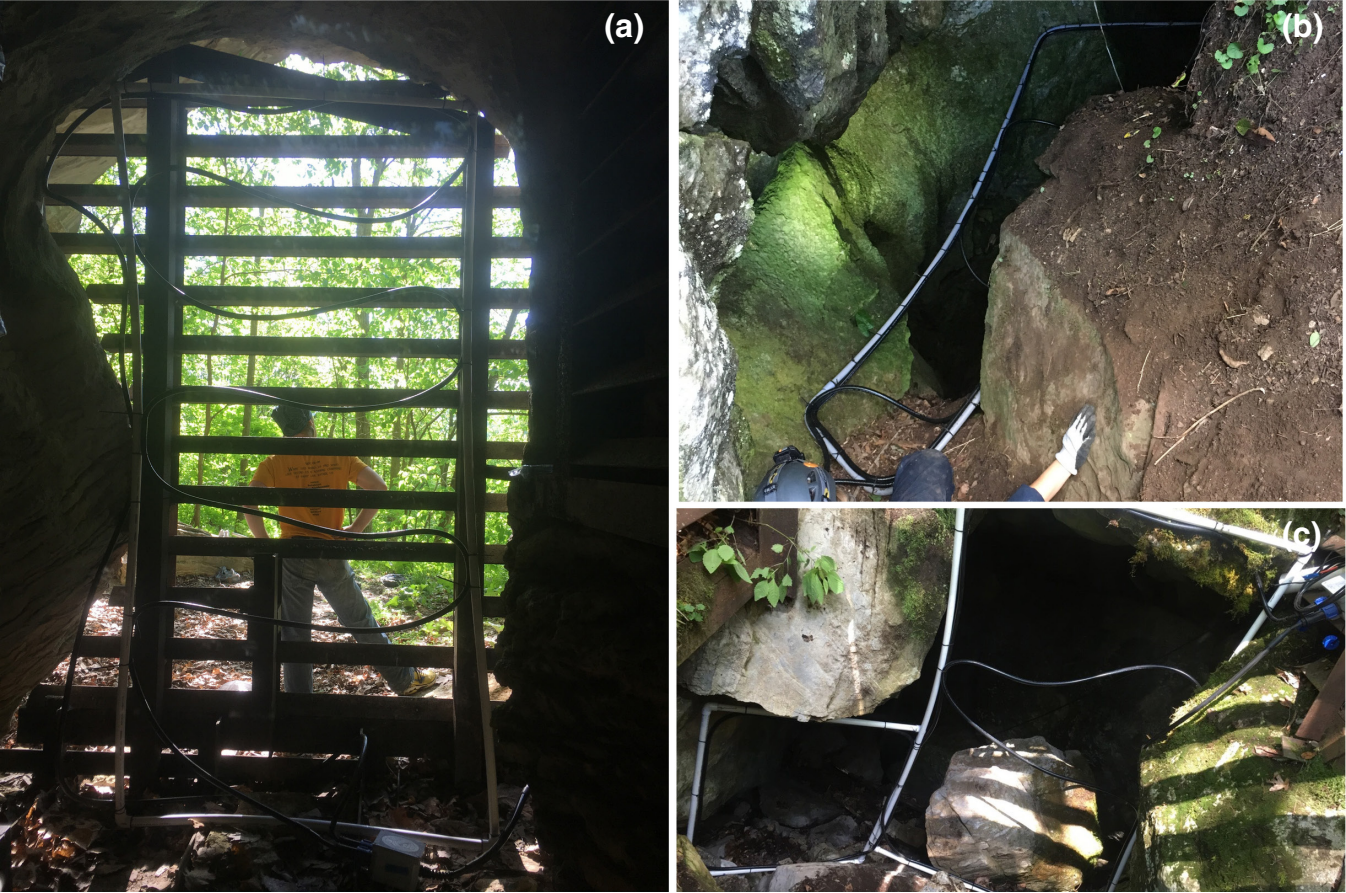
Fifteen‐meter passive integrated transponder (PIT) antennas attached to a PIT‐tag reader/data‐logger (IS1001 Cord Antenna System, Biomark, Inc., Boise, ID) with an external power source (solar panel and car batteries). Each antenna system was constructed in a unique, cave‐specific fashion to increase coverage and decrease obstruction in front of hibernacula entrances. Antennas were used to determine activity patterns of bats at hibernacula during hibernation (November 1 to March 31), 2016–2019. Shown here are (a) Campbell County Cave, (b) Hawkins County Cave, and (c) Blount County Cave

Each cave entrance was surrounded by a 15‐meter‐long PIT‐tag cable antenna attached to a PIT‐tag reader/data‐logger (IS1001 Cord Antenna System, Biomark, Inc., Boise, ID) with an external power source (van Harten et al., 2019). Antennas were arranged to allow bats to pass within 1 m of the antenna at any point of the entrance while not restricting bat flight (Figure 2). We placed PIT‐tag readers outside caves to minimize disturbance during equipment checks and data collection. Data were downloaded from each reader weekly, when possible, and at a minimum bimonthly. Data were then summarized as the number and identity of tagged bats passing through the reader's sensor field each day.

Due to technical difficulties, our PIT‐tag systems did not run continuously throughout hibernation. Therefore, we categorized each night the PIT‐tag reader was running as “functioning.” A “functioning” night was one in which the PIT‐tag system was operational ≥2 hours, beginning at sunset, to record emergence activity from cave entrances (Bernard & McCracken, 2017; Johnson et al., 2012; Reynolds et al., 2017; Schwab, 2014). Given that 86% of nights during hibernation of 2016–2019 (n = 1039/1208) were “functional,” we divided the number of unique bats active per hibernation stage by an estimate of the number of PIT‐tagged bats remaining at each site at the onset of hibernation (~November 1 in Tennessee; U.S. Fish and Wildlife Service, 2007) to determine activity frequency per hibernation stage for each target species. The number of PIT‐tagged bats remaining at each site at the onset of hibernation was determined as the number of PIT‐tagged individuals detected during October (i.e., the end of fall swarm). We used this estimate, instead of the total number of PIT‐tagged bats at each cave, as the latter includes bats that may not hibernate in the cave and does not consider dropped PIT tags (i.e., pit tags that are ejected or lost after insertion) or natural or WNS‐induced mortality over the course of the study (Horton & Letcher, 2008; Johnson et al., 2012).

### 2.5 Data analysis

We divided hibernation into three stages based on average temperature during winter: early hibernation (November 1 to December 15), mid‐hibernation (December 16 to February 15), and late hibernation (February 15 to March 31; Bernard & McCracken, 2017). We calculated the mean midpoint temperature (i.e., the midpoint between the daily high and low temperature) of days when bats were active within hibernation stages using nearest National Oceanic and Atmospheric Administration (NOAA) weather stations. We applied a positive quadratic root transformation to our activity frequency data (Osborne, 2010). To determine differences in activity frequency among species and hibernation stage, we used linear mixed‐effects models using the lme4 package (Bates et al., 2017) in R 3.6.2 (R Development Core Team, 2019), with activity frequency as the response variable and species and hibernation stage as fixed effects. We deemed year as a random effect due to the addition of a new site (Campbell Cave) in the second year of the study. Post hoc tests of the activity frequency model were also conducted in R. 3.6.3 via least‐square means comparisons using the emmeans package (Lenth, 2019) with no adjustment (i.e., Fisher's LSD test).

## 3 RESULTS

### 3.1 Bat capture and tagging

During August–October and April of 2016–2019, we captured 1480 individuals of our four target species. Gray bats accounted for 52.6% of captures (*n* = 779/1480), followed by Indiana bats (27.1%, *n* = 401/1480). Tricolored bats comprised 13.5% of captures (*n* = 200/1480), and eastern small‐footed bats only 6.8% (*n* = 100/1480). We implanted 1346 PIT tags in our four target species (males: 1235, females: 111; Table 1).

**TABLE 1:** Cumulative number of individuals of four bat species implanted with passive integrated transponder (PIT) tags at three cave hibernacula in Tennessee over three fall swarm (August–October) and spring staging seasons (April), 2016–2019

Species	Cave	Cumulative number of PIT tags implanted					
		2016/2017		2017/2018		2018/2019	
		M	F	M	F	M	F
Eastern small‐footed bat (*Myotis leibii*)	Blount	1	0	4	0	5	0
	Campbell	–	–	35	31	41	39
	Hawkins	0	0	0	0	0	0
Gray bat (*Myotis grisescens*)	Blount	0	0	0	0	0	0
	Campbell	–	–	0	0	0	0
	Hawkins	343	15	684	29	684	29
Indiana bat (*Myotis sodalis*)	Blount	185	11	300	13	310	13
	Campbell	–	–	27	3	29	3
	Hawkins	19	2	22	3	22	3
Tricolored bat (*Perimyotis subflavus*)	Blount	11	2	35	7	39	8
	Campbell	–	–	62	9	99	15
	Hawkins	1	0	6	1	6	1
	Total	560	30	1175	96	1235	111

*Note*: Tags were deployed yearly at all caves except Campbell in 2016/2017 and Hawkins in 2018/2019. Antennae were deployed around the perimeter of cave entrances to detect PIT‐tagged bats. Activity registered from bats implanted with PIT tags was used to estimate the frequency of winter activity.

### 3.2 Emergence activity frequency

**FIGURE 3:**
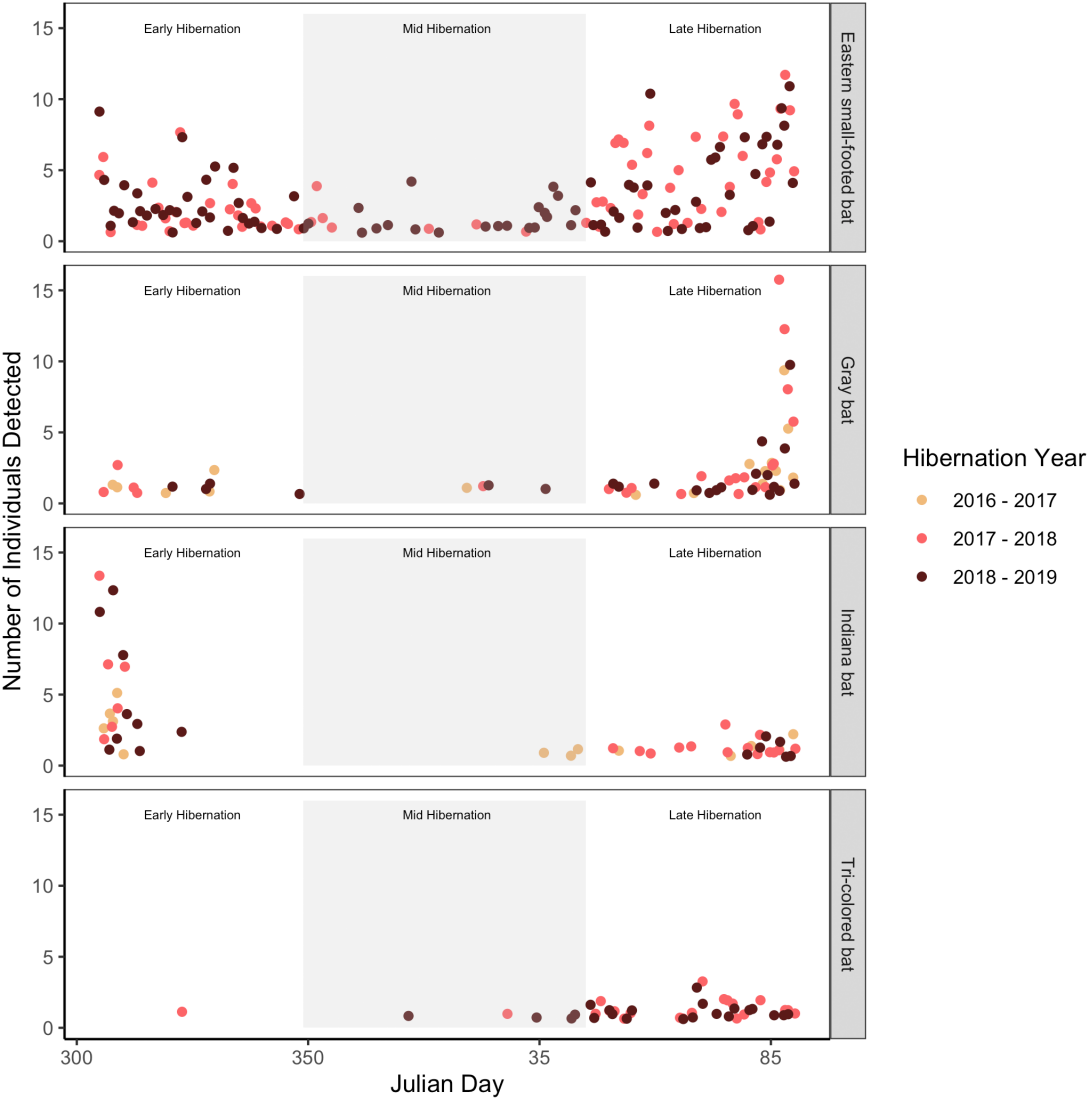
Number of bats implanted with passive integrated transponder (PIT) tags detected daily at three hibernacula entrances in Tennessee during hibernation in 2016–2019. Passive integrated transponder tags were used to track bat movements as they entered and exited hibernacula during three hibernation stages: early (November 1 to December 15, Julian days 305–349), mid (December 16 to February 15, Julian days 350–46), and late (February 15 to March 31, Julian days 47–90)

Over 3 years, 388 individuals of our target species were active from November 1 to March 31, 2016–2019 (Figure 3). Bats were active at hibernacula entrances on days when midpoint temperatures ranged from −1.94 to 22.78°C (mean midpoint temperature = 8.70 ± 0.33°C; Table 2). Eastern small‐footed bats had the greatest frequency of activity during hibernation, with a mean of 83.77 ± 10.90% of tagged individuals detected per hibernation stage. A mean of 29.87 ± 16.22% of tagged tricolored bats were detected across hibernation stages, whereas less than 10% of tagged Indiana bats and gray bats were detected per hibernation stage (Table 3).

**TABLE 2:** Mean daily midpoint temperature (i.e., the midpoint between the daily high and low temperatures) of days with activity from four bat species in Tennessee based on passive integrated transponder (PIT)‐tag detections during hibernation (November 1 to March 31) 2016–2019, per hibernation stage

Daily midpoint temperatures [$\bar{x}$ ± SE]			
Species	Early Hibernation	Mid‐Hibernation	Late Hibernation
Eastern small‐footed bat (*Myotis leibii*)	6.28 ± 0.57°C	7.39 ± 0.79°C	9.01 ± 0.67°C
Gray bat (*Myotis grisescens*)	10.72 ± 1.40°C	9.09 ± 1.09°C	12.12 ± 0.68°C
Indiana bat (*Myotis sodalis*)	14.19 ± 0.97°C	5.74 ± 5.31°C	9.00 ± 1.11°C
Tricolored bat (*Perimyotis subflavus*)	11.67°C	8.28 ± 1.12°C	9.56 ± 0.95°C

*Note*: PIT tags were used to track bat movements as they entered and exited hibernacula during three hibernation stages: early (November 1 to December 15), mid (December 16 to February 15), and late (February 15 to March 31). Temperature data were collected from the National Oceanic and Atmospheric Administration (NOAA) weather station nearest each hibernaculum.

**TABLE 3:** Mean detection rate per hibernation stage of individuals implanted with passive integrated transponder (PIT) tags at entrances of three hibernacula in Tennessee during winter (November 1 to March 31) of 2016–2019

Bat species	Mean % of tagged bats detected; $\bar{x}$ ± SE
Eastern small‐footed bat (*Myotis leibii*)	83.77 ± 10.90%
Gray bat (*Myotis grisescens*)	5.71 ± 2.32%
Indiana bat (*Myotis sodalis*)	7.24 ± 2.49%
Tricolored bat (*Perimyotis subflavus*)	29.87 ± 16.22%

Our linear mixed‐effects models indicated that a species‐by‐hibernation stage interaction best described the frequency of activity throughout hibernation (*p* ≤ .001; Tables 4 and 5). During the early hibernation stage, eastern small‐footed and Indiana bats had significantly higher activity frequencies than gray (*p* < .0179) and tricolored bats (*p* < .0019), with eastern small‐footed bats more frequently active than Indiana bats (*p* = .0033). In mid‐ and late‐hibernation stages, eastern small‐footed bats and tricolored bats had higher activity frequencies than gray (*p* < .0153) and Indiana bats (*p* < .001). However, eastern small‐footed bats were also more frequently active than tricolored bats (*p* = .0221) during mid‐hibernation but not late‐hibernation (*p* = .5543).

**TABLE 4:** Linear mixed‐effects models used to describe the frequency of winter activity of four bat species in Tennessee based on passive integrated transponder (PIT)‐tag detections during hibernation (November 1 to –March 31) 2016–2019. Model parameters show that a Species*Hibernation Stage interaction best described the relationship among species, hibernation stage, and frequency of activity throughout winter

Model	df	*F* value	*p* Value
*Model parameters*			
Species	3	31.8446	<.0001
Hibernation Stage	2	19.0134	<.0001
Species*Hibernation Stage	6	7.5367	<.0001

**TABLE 5:** Mean activity frequency per hibernation stage of individuals from four bat species in Tennessee based on passive integrated transponder (PIT)‐tag detections during hibernation (November 1 to March 31) 2016–2019

Species	Early hibernation	Mid‐hibernation	Late hibernation
Activity Frequency [$\bar{x}$ ± SE]^a^ ^,^ ^b^			
Eastern small‐footed bat (*Myotis leibii*)	100.00 ± 0.00%_A:1_	51.33 ± 14.29%_A:1_	100.00 ± 0.00%_A:1_
Gray bat (*Myotis grisescens*)	2.29 ± 1.42%_A:3_	0.43 ± 0.11%_A:3_	14.41 ± 2.47%_B:2_
Indiana bat (*Myotis sodalis*)	16.23 ± 2.80%_A:2_	0.55 ± 0.55%_B:3_	4.93 ± 0.88%_A:2_
Tricolored bat (*Perimyotis subflavus*)	2.38 ± 2.38%_A:3_	9.96 ± 5.19%_B:2_	77.27 ± 22.72%_C:1_

*Note*: PIT tags were used to track bat movements as they entered and exited hibernacula during three hibernation stages: early (November 1 to December 15), mid (December 16 to February 15), and late (February 15 to March 31).

^a^ $\bar{x}$ ± SE in the same row followed by the same uppercase letter not significantly different (*p* > .05).

^b^ $\bar{x}$ ± SE in the same column followed by the same number not significantly different (*p* > .05).

Within species, eastern small‐footed activity did not differ significantly among hibernation stages (*p* > .1883). Gray and tricolored bats both had significantly higher frequencies of activity during late hibernation than either early (*p* < .0265) or mid‐hibernation (*p* < .0045). However, tricolored bats were the only species that were significantly more active in mid‐hibernation than early hibernation (*p* = .0163). Indiana bats were significantly more active in early and late hibernation compared to mid‐hibernation (*p* < .002), however, there was no difference between early and late stages (*p* = .1073).

## 4 DISCUSSION

Our results indicate that winter activity varies among species, as well as throughout hibernation. As we hypothesized, the eastern small‐footed bat, one of our target species with low susceptibility to WNS, was highly active throughout winter. Gray bats, the other low‐susceptibility species in our study, behaved significantly differently from eastern small‐footed bats, and were relatively inactive outside of caves throughout hibernation. Comparatively, the susceptible species (Indiana bat and tricolored bat) examined demonstrated markedly different activity frequencies throughout hibernation. Tricolored bats, the most susceptible species, exhibited increasingly frequent activity patterns as hibernation progressed. In contrast, Indiana bats, a medium–high susceptibility species, were active on the landscape into early hibernation, after which they did not frequently emerge until late hibernation.

The eastern small‐footed bat (4–6 g) and the tricolored bat (4–10 g), the two smallest species examined in our study, had higher frequencies of activity throughout hibernation when compared to the larger‐bodied gray bat (8–13 g) and Indiana bat (7–10 g). Additionally, the smaller, active species (eastern small‐footed bat and tricolored bat) roost solitarily compared to the larger, less active species that form aggregations during hibernation (Best & Jennings, 1997; Clawson et al., 1980; Fujita & Kunz, 1984; Tuttle, 1979), which may influence their need to arouse from torpor. The large clusters formed by gray bats and Indiana bats during hibernation reduce energy expenditure and total evaporative water loss, which could limit the need for numerous arousals (Barbour & Davis, 1969; Boratynski et al., 2015; Boyles et al., 2008, 2020; Tuttle, 1979). Additionally, data from these sites post‐WNS establishment indicate that these larger‐bodied species have longer torpor bouts than the smaller species examined, with eastern small‐footed and tricolored bats arousing more frequently than Indiana bats (Jackson et al., 2022). Evidence suggests that gray bats may benefit from their inherent behaviors of long torpor bouts in cold temperatures at the low end of the thermal growth optimum for *Pd* (~12.5–15.8°C; Jackson et al., 2022; Verant et al., 2012). In comparison to gray bats, Indiana bats may be suffering the effects of WNS due to their hibernation behavior. Although Indiana bats have long torpor bouts and seem to arouse infrequently, data from this region indicate that the species hibernates at much warmer body temperatures, just outside the upper thermal optimum of *Pd* growth (Jackson et al., 2022; Verant et al., 2012). The medium–high susceptibility exhibited by Indiana bats may be an artifact of long periods spent in torpor in prime *Pd* growing conditions, increasing their chances for *Pd* infection (Langwig et al., 2016).

Arousals from torpor are thought to be catalyzed by both dehydration and energetic needs in bats, making more arousals from torpor necessary for solitarily roosting species dealing with greater energy and water loss, as was observed in eastern small‐footed bats and tricolored bats in our study (Ben‐Hamo et al., 2013; Thomas & Cloutier, 1992). Additionally, starvation and dehydration are thought to be two of the potential causes of mortality in bats suffering from WNS (Cryan et al., 2010, 2013; Willis et al., 2001). Bats affected by WNS exhibit disruptions of various physiological processes that preserve homeostasis, including thermoregulation and water–electrolyte balance, which can lead to respiratory acidosis, hypotonic dehydration, and increased fat metabolism in certain *Myotis* species (Cryan et al., 2010, 2013; Verant et al., 2014; Warnecke et al., 2012, 2013). These physiological interruptions can lead to excessive arousals from torpor throughout winter and may help explain why tricolored bats were found to be increasingly active during the coldest part of winter in Tennessee (i.e., mid‐hibernation). Tricolored bats have fungal loads and prevalence rates that increase substantially as winter progresses (Bernard et al., 2017; Langwig et al., 2016). Our results suggest that the activity we documented was much earlier and more frequent than seen in pre‐WNS hibernation phenology data for this latitude, simultaneously mirroring the seasonal increase in *Pd* in this species (Bernard et al., 2017; Fujita & Kunz, 1984; Langwig et al., 2016; Whitaker & Rissler, 1992). As WNS infections worsen and proliferate, tricolored bats may be exhibiting sickness behavior via increased emergence from hibernacula, thus the activity we documented could have increasingly negative impacts on their survival as winter progresses (Bohn et al., 2016; Langwig et al., 2016; Reeder et al., 2012).

In contrast, pre‐WNS studies have reported regular winter activity of eastern small‐footed bats, suggesting that this behavior is not solely induced by or related to WNS infection in the species (Best & Jennings, 1997; Fenton, 1972; Mohr, 1936). Instead, eastern small‐footed bats have been documented with low *Pd* prevalence and loads throughout their range; therefore, the species may benefit, or at minimum are not harmed, from high levels of activity during winter. Our data indicate that the eastern small‐footed bats examined in our study were active on the landscape throughout hibernation, and only decreased their activity during the coldest period of the year (i.e., mid‐hibernation). Their consistent activity during winter may be correlated with the variety of roost temperatures, humidity levels, and roost types (i.e., talus slopes and rock crevices) used by this species during hibernation, instead of driven solely by disease (Barbour & Davis, 1969; Best & Jennings, 1997; Boyles et al., 2008; Fenton, 1972; Mohr, 1936; Moosman et al., 2015, 2017; Turner et al., 2021). It is possible that eastern small‐footed bats may benefit from this inherent characteristic of their ecophysiology. Bats active throughout winter are repeatedly rising to normothermia, which should activate their immune systems and potentially aid in combating *Pd* invasion (Dobony et al., 2011; Prendergast et al., 2002; Rowley & Alford, 2013; Verant et al., 2014). Additionally, eastern small‐footed bats are known to successfully forage throughout winter, thus increasing their energy intake during hibernation (Bernard et al., 2021). Foraging during hibernation may provide supplemental calories important for surviving the energetic demands of this period, as well as combatting *Pd* infection via increased immune system function (Cryan et al., 2013; Rowley & Alford, 2013; Strandin et al., 2018; Thomas et al., 1990; Verant et al., 2014). Together, the increased caloric intake and immune system function, coupled with low‐level exposure to *Pd* due to roosting preferences, could result in low *Pd* loads seen on eastern small‐footed bats during winter, ultimately sparing them from dramatic declines in populations (Moosman et al., 2017; Turner et al., 2021).

While our results verify that bat species exhibit different levels of winter activity in the southeastern U.S. post‐WNS establishment, it should be noted that our data come from a higher proportion of males than females for three (gray bats, Indiana bats, and tricolored bats) of our four focal species. This is due to the higher number of males captured outside of hibernacula during fall swarming, which is a commonly documented phenomenon of Palearctic cavernicolous bats (Bernard & McCracken, 2017; Lausen & Barclay, 2006; Parsons et al., 2003; Piksa, 2008; Tuttle, 1976; Whitaker & Rissler, 1992). Therefore, our results may reflect a bias toward male behavior in all species except eastern small‐footed bats, where the sex ratio was not skewed. Interestingly, past research at these caves documented no sex biases in other species regularly active throughout winter (Rafinesque's big‐eared bats [*Corynorhinus rafinesquii*] and eastern red bats [*Lasiurus borealis*]; Bernard & McCracken, 2017). Previous studies have hypothesized that this skew at cave entrances may be due to the “thrifty female” hypothesis, whereby females arouse less frequently throughout winter to maintain a body condition that allows them to reproduce successfully in the spring (Jonasson & Willis, 2011). In turn, other studies have proposed that bat populations at hibernacula may be inherently skewed based on their geographic location, with more male‐biased hibernacula farther north and more even ratios in the southern U.S. (Parsons et al., 2003; Whitaker & Gummer, 2000). Regardless, our observations show that the high levels of activity seen in eastern small‐footed bats are common behaviors in both male and female bats.

Ultimately, however, while it has been documented that bats remain active outside of caves during winter regardless of WNS status, at least one of our species, the tricolored bat, may be experiencing the deleterious effects of increased arousal frequency caused by WNS infection late in hibernation. In contrast, the high rate of activity of eastern small‐footed bats may directly benefit this species by indirectly reducing *Pd* loads and associated negative effects. Our findings can help natural resource managers make informed decisions regarding WNS‐affected species, specifically related to timing for when to implement actions (i.e., when vulnerable species are more or less active depending on the type of action) and identifying persistent knowledge gaps related to how and why certain species are maintaining activity in winter. For example, WNS management developed and available for use in hibernacula can be timed to minimize fungal growth and premature emergence of highly susceptible species more effectively. Alternatively, if susceptible species are found to be active throughout winter, managers could develop tools to increase insect availability at hibernacula to supplement food resources with the goal of aiding bat survival through winter. Management efforts targeted at highly susceptible species, versus a broad‐spectrum approach, are likely to be more efficient and less costly, allowing for long‐term management.

Additionally, our data are useful for future research focused on understanding specific details of hibernation and emergence phenology at hibernacula during WNS establishment. Therefore, we encourage future research to use similar methods to investigate the behaviors of gray bats, eastern small‐footed bats, Indiana bats, and tricolored bats in other portions of their range to determine if the trends we observed vary geographically. In doing so, researchers and natural resource managers may better understand how WNS‐affected species may vary in their response to infection across their range and help improve our understanding of the likelihood of persistence of WNS susceptible populations.

## AUTHOR CONTRIBUTIONS

**Reilly Tempest Jackson:** Formal analysis (equal); investigation (lead); methodology (equal); project administration (lead); writing – original draft (lead); writing – review and editing (equal). **Emma V. Willcox:** Conceptualization (equal); funding acquisition (equal); investigation (equal); resources (lead); supervision (lead); writing – review and editing (equal). **John M. Zobel:** Formal analysis (lead); methodology (supporting); supervision (supporting); writing – review and editing (supporting). **Riley F. Bernard:** Conceptualization (equal); funding acquisition (equal); project administration (equal); resources (equal); supervision (equal); writing – review and editing (equal).

## CONFLICT OF INTEREST

The authors declare that they have no conflict of interest.

## Supporting information


Appendix S1

## Data Availability

The datasets generated and/or analyzed during the current study are available in the Figshare repository, 10.6084/m9.figshare.17108261.
