# Supplementary material for: Emergence activity at hibernacula differs among four bat species affected by white‐nose syndrome
Source: Ecol Evol. 2022 Jul 13;12(7):e9113. doi: 10.1002/ece3.9113 (PMC9277409; doi:10.1002/ece3.9113)
Supplement: Supplementary file 1 — Appendix S1 [file ECE3-12-e9113-s001.docx]

**Appendix 1**

Caption: This data provides the methodology and results of five years of disease surveillance efforts at three Tennessee caves where Passive Integrated Transponder (PIT) tags were also deployed. This data is included to provide further evidence for the status of susceptibility to WNS our four target species exhibited throughout the study period.

During the winters of 2012–2014 and 2015–2018, we swabbed 1,144 bats from our four focal species (tri-colored bat, Indiana bat, eastern small-footed bat, and gray bat) for the presence of *Pseudogymnoascus destructans* (*Pd*), the fungal agent that causes white-nose syndrome (WNS). To determine *Pd* load (i.e., infection severity) and prevalence (i.e., number of bats positive for *Pd* over the total number of bats captured and swabbed) of captured bats, we collected fungal samples using a sterile, polyester-tipped epidermal swab dipped in deionized water. We rubbed the epidermal swab across the right forearm and muzzle of each bat five times each (Langwig et al. 2015) and placed the swab in a 2 ml microtube filled with RNAlater® tissue stabilization solution (Life Technologies, Grand Island, NY). Epidermal swab samples were stored at 4°C until they were shipped to the Foster lab at Northern Arizona University and the University of New Hampshire for analysis. All bats were released at the site of capture. While mist netting, followed decontamination procedures outlined by the U.S Fish and Wildlife Service (Shelley et al. 2013). Capture, handling, and sample collection protocols were approved by the University of Tennessee Institutional Animal Care and Use Committee (IACUC 2253-0317), as developed by the American Society of Mammalogists (Sikes et al. 2016) and authorized under scientific collection permits from the USFWS (TE35313B-3), NPS (GRSM-2018-SCI-1253), TWRA (3742) and TDEC (2009-038).

Epidermal swab samples were analyzed using quantitative PCR (qPCR) assays developed by Muller et al. (2013). As per Bernard et al. (2017), they extracted fungal DNA from samples using DNEasy 96 Blood & Tissue kits (Qiagen Inc., Valencia, CA). They tested all samples, as well as negative control wells distributed across each PCR plate, for the presence of *Pd* DNA using a Real-Time PCR assay targeting the intergenic spacer (IGS) region of the rRNA gene complex. They ran all plates in duplicate with a quantified standard of isolate *Pd* 20631-21. They considered any reaction that crossed the threshold baseline in less than 40 cycles on either plate positive for *Pd* DNA. Average *Pd* load in nanograms (ng) was then calculated in each sample based on the cycle threshold (Ct) value and a generated standard curve based on serial dilutions and an equation described by Langwig et al. (2015) and Bernard et al. (2015). We considered a sample to be positive for the fungus (*Pd*+) if at least one of the two replicates had Ct values of less than 40. Otherwise, a sample was considered negative for the fungus (*Pd-*). As our study subjects were active bats, we opted to use a more liberal cut off for positive designation to ensure detection of *Pd* even after extensive activity (i.e., arousal, grooming, and flight; Mosher et al. 2018).

| **Table S1.** Mean loads (log_10_ng) of *Pseudogymnoascus destructans (Pd),* the fungal agent of white-nose syndrome, on bats captured at three cave hibernacula in Tennessee over five hibernation seasons (November 1–March 31), 2012/13−2017/18. | | | | | |
| --- | --- | --- | --- | --- | --- |
| Species | *Pd* Load ($\bar{x}$ + SE) | | | | |
|  | 2012/2013 | 2013/2014 | 2015/2016 | 2016/2017 | 2017/2018 |
| Eastern small-footed bat (*Myotis leibii*) | -4.466 ± 0.123 | -4.747 ± 0.151 | -4.499 ± 0.182 | -4.785 ± 0.170 | -3.988 ± 0.482 |
|  |  |  |  |  |  |
| Gray bat (*Myotis grisescens*) | -5.021 ± 0.086 | -5.196 ± 0.061 | -5.154 ± 0.164 | -5.343 ± 0.000 | -4.803 ± 0.193 |
|  |  |  |  |  |  |
| Indiana bat (*Myotis sodalis*) | -2.642 ± 0.123 | -2.922 ± 0.375 | -2.841 ± 0.219 | -- | -- |
|  |  |  |  |  |  |
| Tricolored bat  (*Perimyotis subflavus*) | -- | -2.189 ± 0.101 | -2.233 ± 0.498 | -3.395 ± 0.535 | -1.695 ± 0.377 |
| --: Bats were not captured/swabbed during this season | | | | | |

| **Table S2.** Prevalence (%) of *Pseudogymnoascus destructans,* the fungal agent of white nose syndrome, on four bat species captured at three cave hibernacula in Tennessee over five hibernation seasons (November 1–March 31), 2012/13−2017/18. | | | | | | |
| --- | --- | --- | --- | --- | --- | --- |
| Species | | Prevalence ($\%$) | | | | |
|  |  | 2012/2013 | 2013/2014 | 2015/2016 | 2016/2017 | 2017/2018 |
| Eastern small-footed bat (*Myotis leibii*) | | 86.67 | 35.29 | 16.13 | 41.67 | 28.57 |
|  | |  |  |  |  |  |
| Gray bat (*Myotis grisescens*) | | 44.44 | 30.77 | 5.88 | 4.17 | 33.33 |
|  | |  |  |  |  |  |
| Indiana bat (*Myotis sodalis*) | | 94.44 | 87.50 | 100.00 | -- | -- |
|  | |  |  |  |  |  |
| Tricolored bat  (*Perimyotis subflavus*) | | -- | 100.00 | 100.00 | 80.00 | 100.00 |
| --: Bats were not captured/swabbed during this season | | | | | |  |
